# Prophylactic Transcatheter Arterial Embolization Helps Intraoperative Hemorrhagic Control for REMOVING Invasive Placenta

**DOI:** 10.3390/jcm7110460

**Published:** 2018-11-21

**Authors:** Kun-Long Huang, Ching-Chang Tsai, Hung-Chun Fu, Hsin-Hsin Cheng, Yun-Ju Lai, Hsuan-Ning Hung, Leo Leung-Chit Tsang, Te-Yao Hsu

**Affiliations:** 1 Department of Obstetrics and Gynecology, Kaohsiung Chang Gung Memorial Hospitaland Chang Gung University College of Medicine, Kaohsiung 83301, Taiwan; MR9221@cgmh.org.tw (K.-L.H.); aniki@cgmh.org.tw (C.-C.T.); allen133@cgmh.org.tw (H.-C.F.); chokovarous@cgmh.org.tw (H.-H.C.); lusionbear@cgmh.org.tw (Y.-J.L.); agneshenrysean@gmail.com (H.-N.H.); 2 Department of Diagnostic Radiology, Kaohsiung Chang Gung Memorial Hospital and Chang Gung University College of Medicine, Kaohsiung 83301, Taiwan; leolctsang@gmail.com

**Keywords:** transcatheter arterial embolization, intrapartum hemorrhage, abnormal placentation, placenta removal, placenta extirpation, cesarean section

## Abstract

Objectives: The purpose of this article is to investigate the estimated blood loss in pregnant women undergoing cesarean section and placental extirpation to treat abnormal placentation and compare the outcomes of those who underwent prophylactic transcatheter arterial embolization (TAE) with those who did not. Methods: A retrospective study was conducted on 17 pregnant women diagnosed with abnormal placentation in 2001–2018 in a single tertiary center. The patients were diagnosed by surgical finding, ultrasound, or magnetic resonance imaging (MRI). These patients were divided into two groups: a prophylactic TAE group (11 patients) and a control group (6 patients). In the former group, prophylactic TAE of the bilateral uterine artery (UA) and/or internal iliac artery (IIA) was performed immediately after delivery of the infant. The placenta was removed in both groups. The primary outcomes were estimated blood loss (EBL), units of packed red blood cell (pRBC) transfusion, operative time, whether hysterectomy was performed, whether the patient was transferred to the intensive care unit (ICU), and hospitalization days. The secondary outcome was maternal complications. Results: Patients who received prophylactic TAE had significantly reduced intraoperative blood loss (990.9 ± 701.7 mL vs. 3448.3 ± 1767.4 mL, *p* = 0.018). Units of pRBC transfusion, operative time, hysterectomy, transfer to the ICU, and postoperative hospitalization days were not significantly different between the two groups. Thirteen patients (9 in the TAE group and 4 in the control group) received a blood transfusion during the operation. Three patients underwent a hysterectomy (1 in the TAE group and 2 in the control group). Five patients were transferred to the ICU (3 in the TAE group and 2 in the control group) for maternal complications or monitoring. In the prophylactic TAE group, 3 patients (27%) had a subsequent pregnancy within the next 5 years. Conclusions: Prophylactic TAE was safe and effective for reducing intraoperative hemorrhage from removing an invasive placenta in patients with abnormal placentation.

## 1. Introduction

In recent decades, the incidence of placenta accreta has risen from 1 in 4027 to 1 in 533 deliveries in the US due to the increased number of patients receiving cesarean sections [1]. According to the invasion depth of chorionic villi, abnormal placental implantations are classified as accreta, increta, and percreta. Although the pathogenesis of abnormal placentation is not yet clearly understood, the most accepted theory is that it results from an imbalance between decidualization and trophoblast invasion. Dysfunctional decidua in a scarred area caused by previous uterine surgery leads to invasion of the myometrium by the placenta [2]. The most common risk factors for invasive placenta are placenta previa and previous cesarean surgery [3]. Placenta accreta is much more common than increta and percreta [4]. 

A precise preoperative diagnosis of abnormal placentation is an important factor for surgical planning. Abnormal placentation is mainly diagnosed by obstetrical sonography in the second or third trimester [5]. Pregnant women with any risk factors (placenta previa, previous cesarean delivery, or uterine surgery) should undergo Doppler ultrasound to investigate the possibility of an invasive placenta. Magnetic resonance imaging (MRI) is suggested when suspected bladder invasion or posterior placenta previa are reported [6]. Compared to an ultrasound, MRI is not routinely used as a screening examination because the sensitivity is less than that of ultrasound (76.9% vs. 100%) and is not cost-effective [7].

The average estimated blood loss (EBL) in women with placenta accreta during delivery is about 3000–5000 mL [8]. The occurrence of massive intraoperative hemorrhage can result from abnormal placentation, maternal morbidity, and mortality increase [9]. Pan et al. concluded that prophylactic intraoperative uterine artery embolization was a safe and effective means for reducing blood loss among patients with an invasive placenta [10]. Besides arterial embolization, prophylactic balloon occlusion of the abdominal aorta, common iliac, internal iliac, or uterine arteries has been reported to minimize intraoperative blood loss [11,12,13,14]. The goal of these procedures is to decrease the uterine perfusion by occlusion of the main supply arteries so that surgical complications and blood loss are reduced. 

Due to the limited number of studies focusing on placenta expulsion in patients with an invasive placenta, the aim of this study is to investigate the efficacy and safety of prophylactic transcatheter arterial embolization (TAE) in patients with abnormal placentation who undergo placenta extirpation.

## 2. Material and Methods

### 2.1. Data Source and Participants

This retrospective study was conducted with 17 patients with abnormal placentation who underwent a cesarean section at the Kaohsiung Chang Gung Memorial Hospital in Taiwan between January 2001 and July 2018. Eleven patients who were treated with prophylactic transcatheter arterial embolization (TAE) were in the prophylactic TAE group, and six patients who did not receive this intervention were in the control group. All patients had placenta previa totalis. Data were collected by reviewing charts of patients who had an invasive placenta and were undergoing cesarean section and placenta expulsion. The diagnosis of abnormal placentation was based on imaging or surgical findings. Placenta previa and invasive placenta were diagnosed by prenatal ultrasound. MRI was not routinely performed, except in cases of suspected placenta increta or percreta. Surgical findings of an invasive placenta included a difficult manual piecemeal removal of the placenta after 20 min and massive rough surface bleeding at the implantation site of a well-contracted uterus [1]. The depth of placental invasion was pathologically confirmed when patients underwent a hysterectomy due to massive postpartum hemorrhage. Our study was approved by the institutional review board (number: 201801593B0). Before performing prophylactic TAE, each patient and her family were informed of the potential risks and completed a consent form.

### 2.2. Interventional Procedures

Prior to the cesarean section, the patients were supine on the operation table. Then, a 5-Fr femoral arterial sheath was inserted into the femoral artery. The procedure took about 5–10 min and did not use a fluoroscope. The neonate was delivered immediately via a classical or transverse cesarean section after general anesthesia. The umbilical cord and bleeding vessels of the uterus were clamped by hemostatic forceps, and a gauze bandage was packed in the uterine cavity. The placenta remained in situ before arterial embolization. The embolization material was gelatin-sponge pledgets (Pharmacia and Upjohn, Kalamazoo, MI, USA), which were cut into 1 × 1 mm and 2 × 2 mm sizes and loaded into 10 mL syringes containing a contrast medium. Under fluoroscopic guidance, a 5-Frangiocatheter (Roberts uterine curve: Cook, Inc., Bloomington, IN, USA) was placed into the bilateral uterine artery (UA) and bilateral internal iliac artery (IIA), and these engorged arteries were embolized with Gelfoam pledgets until a marked slowing of the blood flow was detected by angiography. This procedure took about 30–60 min. The precise time of maternal radiation exposure was not available, because the immediate time record was not transmitted to a central recording system in the portable fluoroscope. The placenta was removed manually after TAE and the arterial sheath was left in situ in case of further unexpected embolization. An emergent hysterectomy was performed if persistent bleeding occurred in patients who had undergone their second session of TAE (lochia ≥ 1000 mL) or had not undergone embolization (EBL ≥ 3000 mL). Patients with complications or just receiving postoperative monitoring were transferred to the ICU for further care. The 5-Fr femoral arterial sheath was removed 48 h later if no active vaginal bleeding was detected and vital signs were stable.

### 2.3. Outcome Measurement, Comorbidities, and Complications

Intraoperative EBL, units of packed red blood cell (pRBC) transfusion, operative time, hysterectomy, transfer to the ICU, and postoperative hospitalization days were the primary outcomes. The secondary outcome was maternal complications, such as pulmonary edema, postpartum hemorrhage, infection, or transient buttock ischemic pain. The Apgar score was retrieved from the medical record, and all neonates were under a pediatrician’s close care.

### 2.4. Statistical Analysis

Continuous variables were evaluated via the Kolmogorov–Smirnov test for distribution. Independent *t*-tests were used for normal distribution variables and their values are described as the mean ± SD. The Mann–Whitney U test was used for non-normal distribution variables and their values are described as the mean ± SD (median, minimum–maximum). Categorical variables were analyzed using the chi-squared test. The statistical analysis program used was SPSS version 22 (SPSS, Chicago, IL, USA), and *p* values of <0.05 were considered statistically significant.

## 3. Results

### 3.1. Characteristics of the Participants

There were 17 patients diagnosed with an invasive placenta who were undergoing placenta extirpation: 11 in the prophylactic TAE group and 6 in the control group. The average age, body mass index (BMI), gravidity, parity, previous cesarean delivery, prior uterine surgery, placental pathology, and gestational age did not significantly differ between the two groups. Transverse cesarean section was performed more often in the control group, and classical cesarean section was carried out more often in the prophylactic group (*p* = 0.035). No significant difference was observed between the groups for neonatal birth weight and Apgar score (<7) at 1 and 5 min (Table 1).

### 3.2. Primary and Secondary Outcomes

Of the primary outcomes, EBL was lower in the prophylactic TAE group (990.9 ± 701.7 mL vs. 3448.3 ± 1767.4 mL), and the difference was statistically significant (*p* = 0.018). Based on placental pathology, for patients with placenta accreta who underwent prophylactic TAE, a lower mean EBL was observed (1055.0 ± 704.9 mL vs. 2133.3 ± 862.2 mL, *p* = 0.048). The EBL was 350 mL in one patient with placenta increta in the prophylactic TAE group, and it was 2300 mL in one patient with the same diagnosis in the control group. There were no patients with placenta percreta in the prophylactic TAE group. There were two patients with placenta percreta in the control group, and their mean EBL was 4645.0 ± 1916.3 mL. No significant difference was observed between the groups for units of pRBC transfusion, operative time, hysterectomy, transfer to the ICU, and postoperative hospitalization days (Table 2).

### 3.3. Complications and Additional Analysis

For the secondary outcome of maternal complications, one patient in the prophylactic TAE group had pulmonary edema and three patients had postpartum hemorrhage (one in the prophylactic TAE group and two in the control group). There was no statistical difference in maternal complications between the two groups (Table 3). All of the patients were transferred to the ICU for further care. One patient in the prophylactic TAE group was transferred to the ICU for monitoring only without experiencing complications. There were no maternal deaths in our study. Of the 11 patients undergoing prophylactic TAE, only one patient with a failed second session of TAE underwent a hysterectomy, which was due to massive postpartum hemorrhage. Embolization of the bilateral uterine artery (UA) and/or internal iliac artery (IIA) occurred in nine (81.8%) patients; embolization of the bilateral UA occurred in only two (18.2%) patients. The two (18.2%) patients in the TAE group with maternal complications had pulmonary edema and postpartum hemorrhage, respectively. Three (27.2%) patients had a subsequent pregnancy within the next 5 years (Table 4).

## 4. Discussion

On the basis of this retrospective study, prophylactic intraoperative TAE significantly reduced the blood loss arising from the removal of the placenta in patients with an invasive placenta. Based on the depth of placental invasion, patients with placenta accreta undergoing prophylactic TAE and placenta extirpation also had significantly less blood loss than those in the control group. However, because of the small numbers of patients with placenta increta or percreta, analysis of EBL in patients with this diagnosis was not possible in this study. No significant difference was observed between the two groups for units of pRBC transfusion, operative time, hysterectomy, transfer to the ICU, and postoperative hospitalization days. The maternal complications of pulmonary edema and postpartum hemorrhage occurred in four patients. The difference in maternal complications between the two groups was insignificant. In the prophylactic TAE group, the clinical success rate was 90.9% and the proportion of patients having both bilateral UA and bilateral IIA embolization was 81.8%. Three (27.2%) patients had a subsequent pregnancy within the next 5 years.

Interventions for hemorrhage control in patients with abnormal placentation include balloon occlusion and arterial embolization with embolic agents. Some physicians express concern that the radiation involved might be harmful to the baby. The harmful dosage of fetal radiation exposure is above 150 milli-Grays, based on the recommendations of the International Commission on Radiological Protection [15]. Currently, whether fetal radiation exposure translates to a risk of childhood cancers remains controversial. Despite prophylactic balloon occlusion reducing intraoperative blood loss, some complications, such as vessel injuries, pseudoaneurysm, thrombosis, bladder necrosis, and neurological damage, have been reported [16]. Since Gelfoam particles dissolve approximately by 3–4 weeks after embolization with returned uterine perfusion [17], the radiologists in our hospital selected this material for prophylactic arterial embolization or postpartum hemorrhage. In our study, all patients in the prophylactic TAE group received bilateral UA and/or bilateral IIA embolization immediately after fetal delivery and before removing the invasive placenta. The mean EBL was significantly lower in the prophylactic TAE group (990.9 ± 701.7 mL vs. 3448.3 ± 1767.4 mL, *p* = 0.018).

Management strategies for an invasive placenta include surgical hysterectomy, conservative treatment—with the placenta left in situ [18]—and removal of the invasive placenta without a hysterectomy. Within the past 10 years, a cesarean hysterectomy has become a common recommendation for patients with placenta accreta [19]. Compared to conservative management, this method reduces maternal complications, but it sacrifices fertility. Conservative treatment of an invasive placenta left in situ preserves fertility, but it poses the risk of potential infection, postpartum hemorrhage, delayed hysterectomy, and maternal morbidity and mortality [20]. Removal of the invasive placenta may prevent the above complications, but it also results in a high risk of massive peripartum blood loss. In order to reduce peripartum blood loss, prophylactic TAE has been promoted by some authors [21]. This approach enables obstetrical surgeons to control intraoperative bleeding with the assistance of a radiologist. If embolization fails or the patient is hemodynamically unstable, hysterectomy is indicated for bleeding control and prevention of progressive complications.

Pan et al. [10] demonstrated significantly reduced mean estimated blood loss in patients with placenta accreta after uterine artery embolization compared to those without the intervention (900 mL vs. 1373 mL, *p* = 005) in a study wherein the placenta was either removed or left in situ. In our study, patients with placenta accreta undergoing placenta extirpation had significantly less blood loss than those in the control group (1055.0 ± 704.9 mL vs. 2133.3 ± 862.2 mL, *p* = 0.048), suggesting it was effective and safe for removing an invasive placenta in patients with accreta to preserve fertility. For patients with placenta increta or percreta, this extirpation management may cause a higher risk of severe bleeding and maternal morbidities. Uteroplacental neovascularization is markedly complex in patients with a deeply invasive placenta, especially in placenta percreta. An extrauterine vascular anastomosis may result in the failure of artery embolization. Consequently, embolization of the uterine artery without other anastomotic arteries is considered to be an insufficient treatment for patients with placenta increta or percreta [22]. In our practice, we preferred to keep the placenta in situ rather than use the extirpation approach for patients with placenta increta or percreta. Two patients with placenta increta and one with placenta percreta were preoperatively diagnosed with placenta accreta. Hence, they underwent a hysterectomy due to massive intraoperative hemorrhage after placenta extirpation.

Major complications associated with TAE are relatively rare, and the complication rate is reported to be about 6–7% [23]. These include post-procedure fever, infection, transient foot or buttock ischemia, iliac thrombosis, and uterine necrosis. No related major complications were documented in our study, but TAE has potential risks. Involvement of a skillful and experienced technician, the size and location of the embolization, and catheter location are important factors to prevent complications. Vedantham et al. [23] believed that ischemic complications of embolization were related to embolic particle size. Gelfoam pledgets, rather than aqueous substances or Gelfoam powder, have the advantage of conferring transient arterial occlusion for hemorrhagic control and recanalization of the target arteries.

From the assessment of fertility and menstrual outcomes, no statistical difference in fertility was observed between patients who underwent embolization and those who did not [24]. However, this study demonstrates a trend toward fewer pregnancies in the embolization group. For patients who desire to preserve their fertility, it is important to inform them before embolization of possible risks and its potential influence on subsequent pregnancy. Lee et al. [25] demonstrated that regular menstruation resumed in 110 (97.3%) of 113 patients who underwent pelvic arterial embolization, while irregular menstruation occurred in two patients, and early menopause due to Sheehan syndrome occurred in one patient. In our prophylactic TAE group, three (27.2%) patients had a subsequent pregnancy and delivered full-term neonates through a cesarean section, while hysterectomy was performed in one patient due to postpartum hemorrhage (PPH) and later outcomes in seven patients were lost in long-term follow-up. TAE appears to be an effective procedure for preserving the uterus, thus enabling resumed regular menstruation and childbearing function, compared to traditional hysterectomy.

A major strength of our study is the compared efficacy of TAE in patients treated with extirpation for an invasive placenta. Due to limited data and the controversy involved with the extirpation approach for an invasive placenta, this retrospective study investigated the efficacy and safety of prophylactic TAE in patients who underwent extirpation for an invasive placenta. The results indicate statistically reduced intraoperative blood loss in the prophylactic TAE group, as well as the preservation of fertility in patients with placenta accreta after the extirpation approach. There are several limitations to our study. First, the sample size was small, particularly in the control group. Second, based on placental pathology, only four patients had placenta increta or percreta. This is not enough statistical power to analyze the efficacy and safety of prophylactic TAE. Third, we had a lack of data on maternal radiation exposure time. The long-term effect of radiation exposure in patients could not be evaluated. Fourth, only four patients (three had a subsequent pregnancy and one had a hysterectomy in 2018) were regularly followed up at our clinics, and the remaining 13 patients were lost in long-term follow-up.

## 5. Conclusions

Many studies have provided evidence on the efficacy and safety of prophylactic TAE for patients with an invasive placenta. However, the question of which is the better management strategy between “extirpation” or “left in situ” for an invasive placenta is currently controversial. We demonstrate the significantly reduced blood loss and successful extirpation management of placenta accreta after prophylactic TAE. However, a larger sample size and long-term follow-up studies are needed to minimize statistical bias. For pregnant women with placenta accreta who desire to preserve their fertility, this approach is considered to be an effective and safe treatment.

## Figures and Tables

**Table 1:** Patient Characteristics.

Characteristic ^a^	Prophylactic TAE Group (*N* = 11)	Non-Prophylactic TAE Group (*N* = 6)	*p* Value
Age (years)	33.8 ± 3.3	34.6 ± 3.3	0.700
Body mass index ^b^	27.1 ± 3.2	24.8 ± 3.5	0.201
Gravidity	1.8 ± 0.75	4.2 ± 2.48	0.053
Prior cesarean deliveries	6(54.5%)	5(83.3%)	0.333
Mode of uterine incision			
Transverse	4(36.4%)	6(100%)	
Classical	7(63.6%)	0	
Prior uterine surgery	2(18.2%)	0	0.515
Placenta type			0.099
Accreta	10(90.9%)	3(50%)	
Increta	1(9.1%)	1(16.7%)	
Percreta	0	2(33.3%)	
Gestational age	35.7 ± 1.56	33.3 ± 4.46	0.542
Neonatal birth weight (g)	2579.5 ± 601.5	2138.3 ± 800.3	0.217
Apgar score < 7			
1 min	4(36.4%)	3(50%)	0.644
5 min	1(9.1%)	3(50%)	0.099

^a^ Values are presented as mean ± SD, or number (%).^b^ Body mass index was calculated as weight in kilograms divided by the square of height in meters.

**Table 2:** Primary outcomes.

Characteristic ^a^	Prophylactic TAE group (*N* = 11)	Non-Prophylactic TAE group (*N* = 6)	*p* Value
Estimated blood loss (mL)	990.9 ± 701.7	3448.3 ± 1767.4	0.018 *
Accreta (mL)	1055.0 ± 704.9	2133.3 ± 862.2	0.048 *
Units of pRBC transfusion	2.9 ± 2.1	6.3 ± 6.9	0.536
Operative time (min)	114.6 ± 21.3 ^b^	148.8 ± 81.9	0.725
Hysterectomy	1(9.1%)	2(33.3%)	0.515
Transfer to ICU	3(27.3%)	2(33.3%)	1.000
Hospitalization days ^c^	7.9 ± 3.3	7.8 ± 3.3	0.913

^a^ Values are presented as mean ± SD, or number (%).^b^ Prophylactic TAE and cesarean section in prophylactic TAE group. ^c^ Postoperative hospitalization. pRBC: packed red blood cell, ICU: intensive care unit. * *p* < 0.05

**Table 3:** Secondary outcome.

Maternal Complications ^a^	Prophylactic TAE Group (*N* = 11)	Non-Prophylactic TAE Group (*N* = 6)	*p* Value
Total	2(20%)	2(33.3%)	0.604
Maternal pulmonary edema	1	0	
Postpartum hemorrhage	1	2	

^a^ Values are presented as number (%)

**Table 4:** Outcomes of TAE group.

Variables	Value
Technical success ^b^	11(100%)
Clinical success ^c^	10(90.9%)
Artery of embolization:	
Bilateral uterine artery and internal iliac artery	9(81.8%)
Bilateral uterine artery	2(18.2%)
No. of patients with complications	2(18.2%)
Subsequent pregnancy	3(27.2%)

^b^ Values are presented as number (%); ^c^ Underwent complete TAE procedure; No postpartum hemorrhage or hysterectomy. TAE: transcatheter arterial embolization.
